# Insensibility Produced by Blue Light

**Published:** 1907-06

**Authors:** 


### INSENSIBILITY PRODUCED BY BLUE LIGHT.

Reard, a professor of dentistry in the University of Geneva, in 1901, first utilized the blue rays of the spectrum for the production of anæsthesia. The technique is given by Cavalie as follows (La Quinzaine therapeutique, March 25, 1907); 1. An electric lamp of 16 candle power, with a glass colored an intense blue and as pure as possible, is employed. 2. The patient should be prepared for a novel experience. He should be reassured and told that he will not be put to sleep, but made insensitive to pain. The method should not be too insistently urged, and if the patient prefers another method the blue light should be given up. The confidence of the patient is an excellent condition of success. 3. The subject is seated in an armchair, of which the back is a little inclined posteriorly. The lamp, furnished with a good reflector, is placed before his eyes at a distance of twelve to sixteen centimetres. 4. The head of the subject and the lamp are lightly covered with a blue cloth (silk or satinet), so as to prevent the diffused daylight from impressing the patient's eyes. 5. The subject should fix his eyes well upon the blue lamp. If an opening be left in the cover it may be closed with a piece of blue glass, through which the face and the eyes of the subject can be watched. 6. Absolute quietness is necessary, no noise in the operating room, and as few words as possible. 7. Two or three minutes of fixation are sufficient. 8. Th subject soon becomes accustomed to the blue color; he complains only of the heat coming from the bulb. Watch the appearance of some light fibrillary movements and dilatation of the pupils. The patient then seems to be in a condition of ecstacy. 9. The insensibility lasts about thirty seconds, and it is necessary then to operate and proceed rapidly. This method suits admirably for the extraction of teeht. It is absolutely without danger. Cavalie has used it in forty cases, almost always with success. In the few cases of failure there was either want of confidence on the part of the patient, or lack of fixation by the eyes upon the blue lamp. According to Renard, failure in 15 or 22 per cent., observed by him, was subject to a similar interpretation. At present the analytical study of the anæsthetizing influence of the blue light has not been carried sufficienly far to permit a scientific interpretation of the results. The reports, however, are very encouraging.—New York Medical Journal, April 27, 1907.

